# An Efficient Deacidification Process for Safflower Seed Oil with High Nutritional Property through Optimized Ultrasonic-Assisted Technology

**DOI:** 10.3390/molecules27072305

**Published:** 2022-04-01

**Authors:** Leyu Xin, Limin Guo, Salamet Edirs, Zepeng Zhang, Chenyang Cai, Yongxing Yang, Yali Lian, Haiyan Yang

**Affiliations:** 1 College of Food Science and Pharmacy, Xinjiang Agricultural University, Urumqi 830052, China; x15739555593@163.com (L.X.); zhangzppeng@foxmail.com (Z.Z.); chenyang.cain@outlook.com (C.C.); x1062815783@163.com (Y.Y.); l1004817296@163.com (Y.L.); 2 Institute of Agro-Products Storage and Processing, Xinjiang Academy of Agricultural Sciences, Urumqi 830091, China; salamet619@163.com; 3 Xinjiang Key Laboratory of Agro-Products Processing and Storage, Urumqi 830091, China

**Keywords:** safflower seed oil, random centroid optimization, ultrasonic-assisted ethanol extraction deacidification, lipid concomitants, antioxidant capacity

## Abstract

Safflower seed oil (SSO) is considered to be an excellent edible oil since it contains abundant essential unsaturated fatty acids and lipid concomitants. However, the traditional alkali-refined deacidification process of SSO results in a serious loss of bioactive components of the oil and also yields massive amounts of wastewater. In this study, SSO was first extracted by ultrasonic-assisted ethanol extraction (UAEE), and the extraction process was optimized using random centroid optimization. By exploring the effects of ethanol concentration, solid–liquid ratio, ultrasonic time, and the number of deacidification times, the optimum conditions for the deacidification of safflower seed oil were obtained as follows: ethanol concentration 100%, solid–liquid ratio 1:4, ultrasonic time 29 min, and number of deacidification cycles (×2). The deacidification rate was 97.13% ± 0.70%, better than alkali-refining (72.16% ± 0.13%). The values of acid, peroxide, anisidine and total oxidation of UAEE-deacidified SSO were significantly lower than those of alkali-deacidified SSO (*p* < 0.05). The contents of the main lipid concomitants such as tocopherols, polyphenols, and phytosterols in UAEE-decidified SSO were significantly higher than those of the latter (*p* < 0.05). For instance, the DPPH radical scavenging capacity of UAEE-processed SSO was significantly higher than that of alkali refining (*p* < 0.05). The Pearson bivariate correlation analysis before and after the deacidification process demonstrated that the three main lipid concomitants in SSO were negatively correlated with the index of peroxide, anisidine, and total oxidation values. The purpose of this study was to provide an alternative method for the deacidification of SSO that can effectively remove free fatty acids while maintaining the nutritional characteristics, physicochemical properties, and antioxidant capacity of SSO.

## 1. Introduction

Safflower (*Carthamus tinctorius*) is an herbaceous and thistle-like annual plant in the sunflower family. It is widely cultivated in Xinjiang, northwest China, for vegetable oil extracted from the seeds. The safflower seed oil (SSO) contains abundant essential unsaturated fatty acids, especially linoleic acid (73–85%), which is ranked number one among the kingdom of oilseed crops [1,2]. SSO is also rich in phenolic compounds, tocopherols, and phytosterols, as the main lipid concomitants, which endows it with great nutritional value and health-promoting functions [3,4]. Polyphenols have outstanding antioxidant and antibacterial effects [5]. In addition, SSO is rich in tocopherols, especially α-tocopherol, valued for their antioxidant properties to protect polyunsaturated fatty acids against oxidation [6]. Safflower seed oil is also a rich source of phytosterols, in particular β-sitosterol and stigmasterol, providing important effects in anti-inflammatory and antitumor activities [7,8]. However, the abundant polyunsaturated fatty acids affect the shelf life of SSO because they are prone to oxidative degradation.

Deacidification, an important refining process in plant seed oil refining, can remove free fatty acids and improve the storage stability of plant seeds oils. Particularly, alkali refining deacidification has been widely used for decades in the edible oil industry [9,10], which is more prone to saponification of neutral oils and causes serious loss of bioactive components, such as sterols and tocopherols among others. Even worse, it generates a massive amount of wastewater. Hence the refining process with alkali deacidification has a drawbacks, and this limits its application in the production of superior-quality edible oils [11]. Ultrasonic-assisted ethanol extraction (UAEE) is performed without alkali addition, at lower operating temperature, and with less processing time comparing to alkali refining, thus it facilitates the retention of lipid concomitants and eliminates the massive wastewater generation [12,13]. Therefore, ultrasonic-assisted ethanol extraction is a preferred method of refining and has high application potential in the production of high-quality plant seeds oils.

Three important indices for the evaluation of the oxidative stability of plant seed oils are acid, peroxide and anisidine values. Acid value is the degree of oil deterioration, reflecting the variety and content of free fatty acids [14,15]. Peroxide value indicates the degree of the initial oxidation products contained in the oil [16]. The products such as lipid hydroperoxides are extremely unstable and easy to further decompose to small molecular compounds such as aldehydes, ketones and acids, etc. The degree of decomposition of those products is usually evaluated using the anisidine value, which represents the degree of deep oxidation of animal and vegetable lipids [17]. Peroxide value and anisidine value are together added up to represent the total oxidation value, which comprehensively evaluates the step-by-step oil oxidation processes [18]. SSO contains a large number of unsaturated fatty acids, which are readily oxidized and become rancid under the ambient environmental conditions of external oxygen content, temperature, light, and other factors. In recent years, many scholars have found that there is a certain relationship between the antioxidant capacity and lipid concomitants of vegetable oil [19,20]. For instance, polyphenols and tocopherols in walnut oil were positively correlated with the free radical scavenging rate, demonstrated that the bioactive components in walnut oil facilitated the antioxidant capacity of walnut oil [21]. Similarly, phenolic compounds in flaxseed oil are closely related to the stability of the oil products due to their antioxidant property [22].

In this study, we aimed to develop an efficient UAEE process for acquiring good-quality SSO. We used deacidification rate as the main index to optimize the UAEE process of SSO by the random centroid optimization (RCO) method. Furthermore, we tested the essential physicochemical properties, fatty acid composition, content of lipid minor components, and in vitro antioxidant capacity of SSO. We also performed a direct comparison for quality parameters of SSO produced by the UAEE and the alkali refining processing method. 

## 2. Results and Discussion

### 2.1. Random Centroid Optimization (RCO)

The method of random-centroid optimization (RCO) is often used in optimizing the ultrasonic extraction process. RCO consists of a random search, a centroid search and a mapping, which constitutes a search cycle. Compared with the commonly used response surface methodology and orthogonal experimental design, RCO is widely applied in the process of optimization for its advantages such as reduced test times and more-accurate optimized parameters [23]. In this study, we investigated four independent factors, including ethanol concentration (%), solid–liquid ratio (*v/v*), ultrasonic time (min), and the number of deacidification cycles. The dependent variable was the rate of deacidification (Section 3).

#### 2.1.1. First-Round Optimization

Conventional methods use an alkaline solution to refine vegetable oil for deacidification and also require a long reaction time and high energy to remove the alkali. These conditions are harsh and often cause degradation and loss of bioactive components of edible oils, in addition to massive amount of wastewater generation. Therefore, it is imperative to develop and use an alternative refining technique to produce high-quality SSO with conserved phytonutrients and extended shelf life. In this study, the UAEE method was evaluated and selected to optimize the deacidification conditions to effectively remove free fatty acids while maintaining the quality of SSO. The RCO experimental methodology was applied in this optimization process of deacidification of SSO. The experimental results of random and centroid search in the first round of optimization are shown in Table 1.

**Figure 1:**
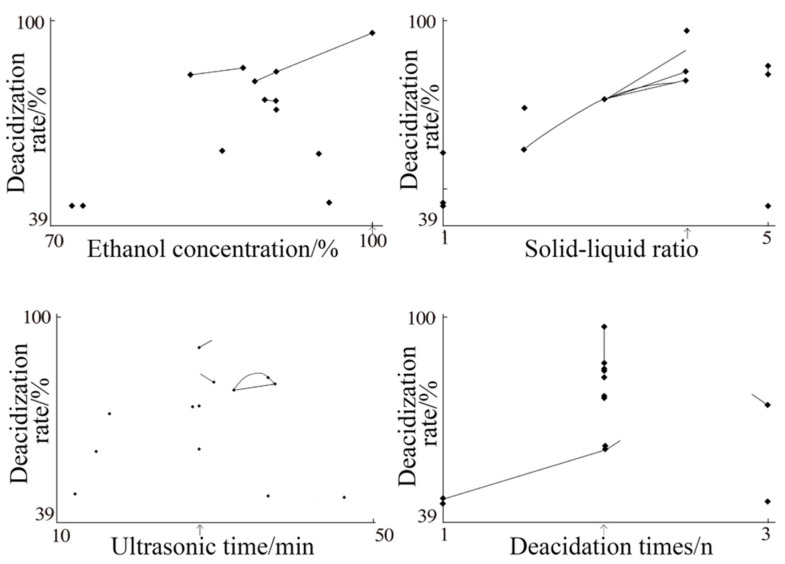
Mapping of the conditions from the first-round optimization.

The mapping optimization was carried out according to the experimental results of random search and centroid search. The mapping optimization diagram from the first round of RCO was obtained, and the specific results are shown in Figure 1, in which each factor was selected in accordance with a mapping optimization diagram.

The position indicated by the abscissa arrow was the best value of this factor. It was preliminarily judged from the position indicated by the arrow in the mapping diagram of the first round of optimization conditions. The optimization results of the deacidification process were as follows: ethanol concentration (100%), solid–liquid ratio (1:4), ultrasonic time (29 min), and number of deacidification cycles (two). However, due to the scattered mapping results in the figure, these were insufficient to reflect the optimization results, hence a second round of optimization was needed to obtain a more reliable optimization process.

#### 2.1.2. Second-Round Optimization

Based on the centroid range and results of the first round of optimization method, the upper and lower limits of factors in the second round of the cycle were inputted, and the second round of the experimental scheme was obtained. The corresponding experimental results are shown in Table 2.

**Figure 2:**
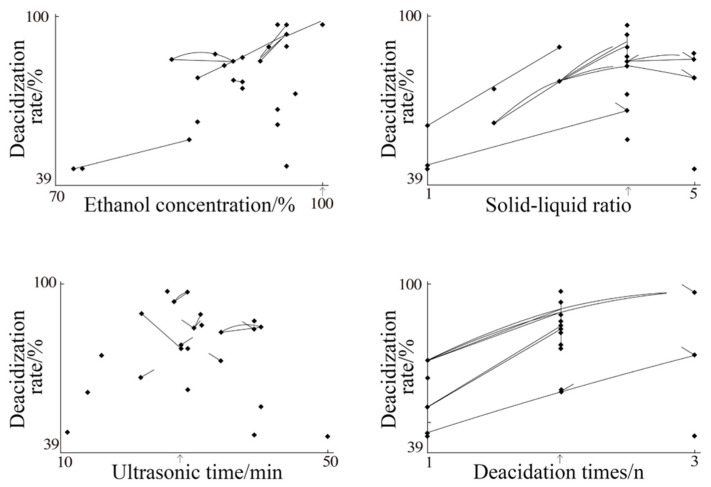
Mapping parameters of the second round of optimization.

According to the above experimental results (Table 2), the mapping optimization was carried out and the mapping optimization diagrams from the second round were obtained. The specific results are shown in Figure 2.

It can be seen from Figure 2 that, compared with the first round of mapping optimization, the midpoint of the map after the second round of optimization is more concentrated and mostly distributed at the position of the arrow, which proves that the results of this round of optimization are reliable. The optimum parameters for the extraction process were as follows: ethanol concentration, 100%; solid–liquid ratio, 1:4; ultrasonic time, 29 min; and 2 cycles of deacidification. This result is consistent with the optimal extraction conditions of the optimization results of the first round of random experiments.

### 2.2. Validation Test

The above optimized process was used to verify the deacidification of SSO. The optimized conditions were 100% ethanol concentration; a ratio of 1:4 for solid to liquid; 29 min of ultrasonic time; and 2 cycles of deacidification, and the final deacidification rate obtained was 96.39% ± 0.05%. The verified results were similar to the measured results, and the standard deviation between the two methods was 0.55. Therefore, the conditions for the optimal extraction process as per RCO met the requirements.

### 2.3. Effect of Deacidification Method on Physicochemical Quality of Safflower Seed Oil

With the alkali-refining process as the control, the UAEE process was used to deacidify SSO. The effects of the different deacidification processes on the physical and chemical quality of SSO were compared. The results are shown in Table 3.

Table 3 shows that both deacidification processes can significantly reduce the acid value of safflower seed oil (*p* < 0.05). The acid value of SSO could be reduced to 0.10 ± 0.01 mg/g by the UAEE process, and the deacidification rate reached 97.13% ± 0.70%, which was better than that by alkali refining deacidification process (72.16% ± 0.13%). The peroxide value of SSO after deacidification by UAEE was 0.96 ± 0.00 mmol/kg, which was significantly lower than that by alkaline refining deacidification (1.50 ± 0.09 mmol/kg) (*p* < 0.05). Compared with safflower seed crude oil, the anisidine value of SSO after alkali refining deacidification waas elevated significantly elevated (1.00 ± 0.04) (*p* < 0.05). Compared with the alkali refining process, the value of anisidine in UAEE deacidification decreased significantly (*p* < 0.05). The total oxidation value of safflower seed oil by UAEE was lower than that of SSO from alkali refining deacidification (3.99 ± 0.15). Acid, peroxide, and anisidine values are all important indexes to evaluate the quality, such as deterioration and oxidation degree, of vegetable oil. The test results showed that the values of these three parameters for SSO deacidified by the UAEE process were significantly better than those for alkali refining deacidification. Due to the higher operating temperature and longer processing time, these three indexes of SSO had a more rapid increase in alkali refining deacidification than in the UAEE method, meaning shorter shelf-life due to more susceptiblity to oxidation and deteriation of the SSO from alkali deacidification process.

### 2.4. Effect of Deacidification Methods on Fatty Acid Composition of Safflower Seed Oil

The composition of major fatty acids and the content of safflower seed oil after different deacidification processes are shown in Table 4.

Linoleic and linolenic acids are both precursors of polyunsaturated triglycerides from omega-3 and omega-6 fatty acids, which are essential for human health and disease prevention [24]. It is seen from Table 4 that the levels of linoleic and linolenic acids were significantly higher after the UAEE process than those after alkali refining. The ratio of oleic and stearic acids to total fatty acids did not change significantly after UAEE treatment but increased significantly after alkali refining. The percentage of palmitic acid showed no difference after deacidification by the two methods. 

### 2.5. Effect of Deacidification Method on the Quality of SSO Concomitants

Crude safflower seed oil was deacidified by alkali refining and by the UAEE process at the same time for comparison. The effects of different deacidification processes on the quality of lipid concomitants in SSO were tested, analyzed, and compared. The results are shown in Table 5.

It can be seen from Table 5 that the loss of α-tocopherol of SSO due to alkali refining deacidification was 17.5% (32.66 mg/kg), much higher than the loss from the UAEE deacidification (5.2% or 9.66 mg/kg), indicating that the UAEE method is superior to alkali refining in conserving α-tocopherol. Furthermore, both deacidification processes caused a loss of total polyphenol content in safflower seed oil, but the polyphenol content in SSO after UAEE deacidification was 9.12 mg/kg, which was significantly higher than that of alkali refining deacidification (7.25 mg/kg) (*p* < 0.05). After the UAEE process, the sterol content in SSO was 66.00 ± 1.00 mg/kg, 12.5% higher than that of SSO (58.67 ± 1.53 mg/kg) after alkali refining.

Tocopherols, polyphenols, and sterols are important lipid componets in SSO. They are widely distributed in plant-based oils, particularly in seed oils, and as part of the lipid components, they are extracted into SSO through pressing and extraction with or without solvents such as alcohols or supercritical fluids [25]. However, due to their heat-labile and oxygen-sensitive nature, they are prone to oxidative decomposition and then polymerization at higher temperature and, hence, are readily lost in the refining process [26]. Compared with the alkali refining method, the operating temperature in the UAEE method is much lower, which significantly reduced the loss of tocopherol, sterol, and polyphenol in SSO in the deacidification and refining process of UAEE (*p* < 0.05). 

### 2.6. Effect of Deacidification Method on Antioxidant Activity of SSO In Vitro

The antioxidant activity of SSO comes from multiple factors, in particular, from many antioxidant components, such as phenolic compounds, tocopherols, and sterols among others. Due to different mechanisms, two testing methods, i.e., 2,2-diphenyl-1-picrylhydrazyl (DPPH) and 2,2′-azinobis (3-ethylbenzothiazoline-6-sulfonic acid) (ABTS), were used to detect the antioxidant activity of SSO in this study. The two methods reflect different aspects of antioxidant properties. The ABTS assay is suitable for evaluating the activity of lipophilic and hydrophilic antioxidants in samples, while the DPPH assay is based on free radicals dissolved in organic media [27,28]. The alkaline refining and UAEE processes were used to deacidify crude SSO, and the effects of different deacidification processes on the antioxidant activity of SSO in vitro were compared. The results are shown in Table 6.

As shown in Table 6, the antioxidant activity according to the DPPH radical scavenging capacity of SSO after UAEE was 35.30 ± 0.28 μmol TE/100 g, which was not significantly different to that of crude oil. However, the result of DPPH after alkali refining deacidification was 32.11 ± 0.28 μmol TE/100 g, which was significantly lower than that of the crude oil (35.76 ± 0.09 μmol TE/100 g) and the UAEE method (*p* < 0.05). One possible explanation for these discrepancies was the presence of more conserved tocopherols, phenolic compounds, and sterols because of the excellent correlation between tocopherols, total phenols content (r = 0.911), and sterols content (r = 0.934) (Table 5).

Compared with crude SSO, the ABTS radical scavenging capacity of the two deacidified SSO oils decreased significantly (*p* < 0.05), but the ABTS value of SSO after UAEE (14.19 ± 0.01 μmol TE/100 g) was higher than the ABTS value of SSO after alkaline refining deacidification (13.93 ± 0.12 μmol TE/100 g). Compared with the DPPH assay, the correlation of ABTS values for SSO samples with their total phenol values (r = 0.932) was better than that of DPPH (r = 0.911). Compared with alkali refining deacidification, the UAEE process improved the antioxidant activity by retaining more antioxidant compounds of SSO such as tocopherols, phenolics, and phytosterols.

### 2.7. Correlation Analysis

#### 2.7.1. Correlation Analysis of Physical and Chemical Quality of SSO

Pearson bivariate correlation analysis was conducted on the three main lipid concomitants of SSO, i.e., α-tocopherol, phenolics, and sterols, and their basic physicochemical qualities in oil samples of crude SSO, UAEE-deacidified SSO, and alkali-refining-deacidified SSO. The results are shown in Table 7.

As shown in Table 7, the three lipid concomitants in safflower seed oil were negatively correlated with values of lipid peroxidation, anisidine, and total oxidation, among which α-tocopherol was negatively correlated with lipid peroxidation value, anisidine value, and total oxidation value, but there was no significance. There was a significant negative correlation between total phenols and anisidine value (*p* < 0.05), suggesting that total phenols contributed greatly to the inhibition of late-stage oxidation of SSO. There were also significant negative correlations between total sterols and lipid peroxidation value (*p* < 0.05) and with anisidine value (*p* < 0.01), indicating that the content of sterols was significantly correlated with the physicochemical quality of SSO at different oxidation states. This may be because, in the early stage of oil oxidation, the sterols acted as reduction substrates and inhibited the oil’s deterioration to a certain extent [29]. According to the results of the correlation analysis, the content of α-tocopherols, total phenolics, and total sterols are closely related to the physicochemical quality of SSO. They are the main components contributing to inhibiting the oxidation of SSO.

#### 2.7.2. Correlation Analysis between Safflower Seed Oil Content and Antioxidant Capacity In Vitro

Pearson bivariate correlation analysis was performed between the free radical scavenging capacity and main lipid concomitants of SSO refined by different methods. The results are shown in Table 8.

Table 8 shows that α-tocopherol in SSO was positively correlated with ABTS and DPPH radical scavenging capacity, but there was no significant difference between the two assays. The correlation analysis showed that there was a significant positive correlation between the total content of phenols and ABTS radical scavenging capacity (*p* < 0.01) and also a significant positive correlation between the phenolic content and DPPH radical scavenging ability (*p* < 0.05). Hence, the phenolic compounds contributed greatly to the radical scavenging effect in both ABTS and DPPH assays, consistent with the reported results by Liu et al. on the correlation between the level of polyphenol content in the methanol extract of rice bran oil and the free radical scavenging ability determined [30].

Furthermore, it can be seen from Table 8 that there is a significant positive correlation between the total content of sterols and the value of ABTS (*p* < 0.05) and also between the sterol content and DPPH values (*p* < 0.01), indicating that sterols in SSO play important roles in DPPH and ABTS assays. Tocopherol is a natural antioxidant in vegetable oil. It can react with lipid free radicals and finally form lipid–tocopherol-derived compounds with high stability. Plant polyphenols can not only directly inhibit oil oxidation by scavenging free radicals but also indirectly inhibit oil oxidation by protecting endogenous antioxidant enzymes [31]. Phytosterol is widely used as a natural antioxidant of oil because of its strong reducibility and free radical scavenging ability for inhibiting oil oxidation [32]. The results of correlation analysis showed that the main lipid bioconcomitants of SSO such as tocopherols, total phenols, and sterols were closely related to the in vitro antioxidant activity of SSO. It is attested that they play significant roles as natural antioxidants in safflower seed oil.

## 3. Materials and Methods

### 3.1. Materials and Chemicals

Safflower seed crude oil was gifted from Yaquina Agricultural Development Co., Ltd. (Yining, China). α-Tocopherol and stigmasterol (standard, ≥95%) were purchased from Sigma-Aldrich (Shanghai, China); 2,2′-azinobis (3-ethylbenzothiazoline-6-sulfonic acid (ABTS, ≥98%), 1,1-diphenyl-2-picrylhydrazyl (DPPH, ≥98%), and Trolox (≥98%) were purchased from Yuanye Biotechnology Co., Ltd. (Shanghai, China). Folin–Ciocalteu reagent was purchased from Solarbio Technology Co., Ltd. (Beijing, China); petroleum ether, isopropanol, glacial acetic acid, isooctane, absolute ethanol, sodium hydroxide, methanol, *n*-hexane, and other chemical reagents used were of either analytical or chromatographic grade and were purchased from Zhiyuan Chemical Reagent Co., Ltd. (Tianjin, China).

### 3.2. Ultrasonic-Assisted Ethanol Extracton

Two methods were employed in this study to develop and optimize the efficiency and quality of the SSO extracted. UAEE and alkali refining deacidification were performed in parallel to compare the contents of extracted antioxidants and other bioactive compounds such as phenolic compounds, phytosterols, and tocopherols.

The extraction process of SSO with UAEE was performed in a SB-520DT ultrasonic cleaning device (Ningbo Scientz Biotechnology Co., Ltd., Ningbo, China). The operating procedure was as follows: 100 mL of crude safflower seed oil was degummed and dehydrated. The resultant residue was weighed and mixed with different concentrations of ethanol in water (*v/v*). The formed solution was then placed in an ultrasonic device for ultrasonic-assisted extraction with a varying number of extractions and time periods for each extraction. When finished, the mixed solution was centrifuged at 4000 r/min for 10 min. The above operation was repeated until the free fatty acid was almost removed. The extracted oil, at the bottom of the liquid solution, was collected, and transferred to a round bottom flask for the subsequent removal of solvent residue in vacuo in a rotary evaporator device.

### 3.3. Deacidification of SSO with Alkali Refining

Alkali refining deacidification was performed according to the method described by Ying et al. with minor modifications [11]. The degummed oil was heated to 65 °C, then alkali aqueous solution was added while stirring. When the oil and soapstock was separated, the stirring was stopped, and the upper oil layer was separated after centrafuge. The alkali-treated SSO was transferred to a separating funnel and washed with deionized water until the discharged water was neutral. Finally, the collected SSO oil was dehydrated to yield alkali-refining-deacidified oil.

### 3.4. Random Centroid Optimization (RCO)

The effects of different factors on the deacidification of SSO were evaluated using RCO method. This universal optimization method is particularly useful for investigating multiple factors and targets and is currently used in the process optimization of food product development. In this study, the deacidification conditions and the range of parameter values were in accordance with those in the related literature [13,33]. The influencing factors were varied as follows: aqueous ethanol concentration, 70–100%; solid–liquid ratio, 1:1–1:5 (*w/v*); ultrasonic time, 10–50 min; and 1–3 cycles of deacidification, as shown in Table 9. The four variables and the ranges of their values were inputted to the RCO program (RCOPTNS), and 13 sets of proposed paramter values were yielded from the random search. These sets of parameters were then applied to the treatment of SSO samples in the UAEE process.

After the SSO treament, the parameter values from the UAEE process were determined and then inputted to the program of the centroid search for further optimized sets of parameter values. This round of centroid search proposed 11 sets of conditions for further experiments. These new sets of parameters’ values were further evaluated and the resultant values were re-inputted into the random search program. Finally, the obtained results were compiled and mapped for directing the optimum UAEE conditions. The extraction conditions of SSO using UAEE process were applied in triplicate following the results from mapping, and their output was then compared with that of traditional alkali refining.

### 3.5. Fatty Acid Analysis

The fatty acid compositions of the extracted SSO were determined by gas chromatography and mass spectrometry (GC/MS, GC 7890A, MS 5975C, Agilent, Santa Clara, CA, USA). The GC/MS conditions are detailed below. The carrier gas used for GC-FID analysis was hydrogen with a constant flow rate of 3.7 mL/min. The injector was maintained at 270 °C; capillary column, polydicyanopropylsiloxane strong polar stationary phase; column length, 100 m; inner diameter, 0.25 mm; and film thickness, 0.2 μm. The detector temperature was 280 °C. The split ratio was 1:100. The oven temperature was kept at 100 °C for 13 min, then programmed to rise to 180 °C at the speed of 10 °C/min and was kept for 6 min; then it rose to 200 °C at the speed of 1 °C/min and was kept for 20 min, and finally it rose to 230 °C at the speed of 4 °C/min and was kept for 10.5 min.

### 3.6. Determination of Acid Value, Peroxide Value, and Anisidine Value

The standards of the ISO (International Organization for Standardization) were used for the determination of acid value (ISO 660, 2009), peroxide value (ISO 3960, 2007), and anisidine value (ISO 6885, 2006). The total oxidation value was calculated according to the following Equation (1):

(1) Total oxidation value=2×POV+p−AV

where *POV* is the peroxide value, mmol/kg; *p − AV* is the anisidine value.

The deacidification rate of safflower seed oil was obtained by the following Equation (2):

(2) $$\mathrm{Deacidification}\;\mathrm{rate}=\frac{S_{1}-S_{2}}{S_{1}}\times 100\%$$

where *S*_1_ is the acid value of the oil sample prior to deacidification, mg/g; *S*_2_ indicates the acid value after deacidification, mg/g.

### 3.7. Determination of Lipid Concomitants

#### 3.7.1. α-Tocopherol

The content of α-tocopherol was determined using the Agilent 1260 Infinity HPLC system (Agilent Technologies Co. Ltd., Palo Alto, CA, USA) according to the method described by Gama et al. [34]. The HPLC system conditions were as follows: diamonsil-C18 column (4.6 mm × 250 mm, 5 μm); UV wavelength, 295 nm; mobile phase, methanol in isocratic elution; injection volume, 20 μL; column temperature, 30 °C; and flow rate, 1.0 mL/min. Peaks were quantified by area compared to the α-tocopherol standard (1.0 mg/L). The curve equation was y = 134,491x + 417,039, R^2^ = 1.0000. α-Tocopherol was isolated from the oil sample as follows: methanol was added to 1.0 g of safflower seed oil with a solid–liquid ratio of 1:1.25; vortex oscillation time, 2 min; ultrasonic extraction, 20 min; and then centrifugation at 4000 rpm for 5 min. The supernatant was collected. This operation was repeated three times. The supernatant lyes were combined, concentrated to dryness in vacuo. Subsequently, 1 mL of methanol was added to the residue, mixed, and filtered through a 0.22 μm filter membrane, which was then analyzed on an HPLC system.

#### 3.7.2. Determination of Total Phytosterol Content

The determination of the total sterol content in SSO was performed according to the following method after a minor modification from Yu et al. [35]. An SSO sample (2.0 g) was added to 6 mL of 2 M potassium hydroxide in 95% ethanol aqueous solution. The resultant solution was mixed and heated to 60 °C for 60 min for saponification. A mixture of n-hexane with deionized water (3:2, *v/v*) was then added, mixed well, and centrifuged at 4500 rpm for 5 min. The supernatant was isolated and concentrated to dryness in vacuo, and then mixed with 2 mL of absolute ethanol and refrigerated at 4 °C for use. An aliquot of the above solution (0.1 mL) was mixed with 2 mL of phosphorus ion reagent (PS-FE reagent) and 2 mL of ethanol, and then measured at 520 nm using a Shimadzu UV2000 Spectrophotometer (Shimadzu, Kyoto, Japan).

The standard curve of stigmasterol was prepared as follows: Stigmasterol (100 μg/mL) in ethanol was prepared as a stock solution. Aliquots of 0.0, 0.5, 1.0, 1.5, 2.0, and 2.5 mL of the stock stigmasterol ethanol solution were transferred to six volumetric flasks, respectively. Then, 2 mL of PS-FE reagent was added to each of the flasks, which were filled with absolute ethanol to a total solution volume of 6 mL. The absorbance value of each resulted solution was mearsured with UV at 520 nm wavelength. The corresponding standard curve equation was obtained as y = 0.001x − 0.006, (R^2^ = 0.9952), in which y is the concentration and x is the absorbance value of a stigmasteral solution.

#### 3.7.3. Determination of the Total Content of Phenolic Compounds

The total phenolic content of SSO was measured according to the method described by Durmaz [36] with slight modifications. Briefly, 1.5 g of each extract was dissolved in 1.5 mL of hexane and extracted with 1.5 mL of methanol aqueous solution (80%, *v/v*) three times. The aqueous layers were combined and centrifuged at 3000 rpm for 10 min. Supernatant of the centrifuged sample was collected. A 500 μL solution of the extract was transferred to a 10 mL calibration flask. Folin–Ciocalteu reagent (0.5 mL) and 2 mL of saturated sodium carbonate solution (4%) were also added to the calibration flask, shaken for 3 min, and then stored in the dark for 1 h. The measurement was carried out at 760 nm using a Shimadzu UV2000 Spectrophotometer (Shimadzu, Kyoto, Japan). Gallic acid was used as the reference standard. Each sample was analyzed in triplicate. Calibration curves of gallic acid were obtained using the least-squares method, resulting in the equation y = 0.0211x + 0.0076 (R^2^ = 0.9998), with a concentration range of 10–60 μg/mL. The results were expressed as mg gallic acid equivalents/kg (mg GAE/kg).

### 3.8. Determination of Antioxidant Activity In Vitro

The antioxidant activity of the extracted SSO was evaluated using two assays: 2,2-diphenyl-1-picrylhydrazyl (DPPH) radical scavenging assay, and 2,2′-azinobis (3-ethylbenzothiazoline-6-sulfonic acid) (ABTS) radical scavenging assay according to the method described by Ncab et al. [37] with slight modifications. Each sample was prepared by dissolving 5.0 g of SSO in 5.0 mL of methanol, which was then centrifuged at 4500 rpm for 5 min, and the supernatant (methanol solution) was collected. The extaction was repeated two more times. The supernatant was combined and refrigerated at 4 °C for the antioxidant assays. This extraction process was repeated in triplicate to obtain three methanol extracts (supernatant) from each SSO sample.

#### 3.8.1. Determination of DPPH Radical Scavenging Capacity

A 2.0 mL sample of supernatant (prepared as described above) from each sample was mixed with 2.0 mL of a DPPH solution (0.1 mmol/L) in methanol and kept in a dark at room temperature for 30 min. The decrease in absorbance was measured at 517 nm on a Shimadzu UV2000 Spectrophotometer (Shimadzu, Kyoto, Japan). Trolox was used as standard and the results were expressed in μmol Trolox equivalent/100 g of SSO (μmol TE/100 g). Each sample was measured in triplicate.

#### 3.8.2. Determination of ABTS Radical Scavenging Ccapacity

The ABTS assay was performed in this study, and the ABTS free radical solution was prepared as following. A solution of ABTS (7.0 mM) was mixed with a solution of potassium persulfate (K_2_S_2_O_8_, 2.4 mM), and the mixed solution was kept in the dark for 12–16 h. The mixed solution was then diluted with methanol (1:1, *v/v*) at pH 7.4 until an absorbance of 0.7 at 734 nm was reached steadily. 

Then, 100 μL of a methanol extract (supernatant) from each SSO sample was mixed with 1.0 mL of ABTS free radical solution and 1.0 mL of methanol in the dark. After 6 min, the absorbance was measured by a Shimadzu UV2000 Spectrophotometer (Shimadzu, Kyoto, Japan). Trolox was the standard reference, and results were presented in μmol Trolox equivalent/100 g of SSO (μmol TE/100 g). Each sample of a methanol extract was measured in triplicate.

### 3.9. Statistical Analysis

Each group of experiments was repeated three times. The results are shown as mean ± SD (standard deviation). ANOVA significance analysis and Pearson correlation analysis were carried out using SPSS 16.0 (IBM, Armonk, NY, USA).

## 4. Conclusions

In this study, ultrasonic-assisted ethanol extraction (UAEE) technology was for the first time applied to the refining deacidification of safflower seed oil. The optimization of the UAEE process was performed with a random centroid optimization program (RCO). The optimum UAEE conditions obtained were as follows: ethanol concentration, 100%; solid–liquid ratio, 1:4; ultrasonic time, 29 min; and 2 cycles of deacidification. The safflower seed oil was rich in unsaturated fatty acids, mainly linoleic acid, and bioactive compounds such as polyphenols and phytosterols. Compared with alkali refining deacidification, the UAEE method effectively retained bioactive compounds, improved the physicochemical properties of the safflower seed oil, and enhanced antioxidant capacity. Overall, crude SSO after the UAEE process showed desirable physicochemical characteristics, satisfactory amounts of bioactive compounds, and good in vitro antioxidant capacity. Therefore, the UAEE-processed SSO may be a good source of edible oils, and the UAEE method appeared to be preferably suitable for deacidification of safflower seed oil.

Furthermore, alkali refining is a traditional deacidification process widely used currently in practical industrial production. However, due to its high operating temperature, high refining energy consumption, massive generation of waste water, and great loss of bioactive components, a new method is urgently needed to overcome the drawbacks in SSO processing using alkali refining. The UAEE process developed in this study has the advantages of low operating temperature, short treatment time, and high retention rate of bioactive ingredients. Thus, it has the advantages of being fast, economical, and environmental friendly. Therefore, UAEE has a broad application prospect.

## Figures and Tables

**Table 1:** Results of the first round of optimization experiment.

Step	Experiment No.	Ethanol Concentration (%)	Solid–Liquid Ratio	Ultrasonic Time (min)	Number of Deacidification Cycles	Deacidification Rate (%)
Random search design	1	86	1:2	29	2	61.59 ± 0.70
	2	88	1:5	39	2	86.32 ± 0.06
	3	73	1:1	39	3	45.21 ± 0.26
	4	96	1:1	11	1	46.00 ± 0.39
	5	95	1:1	14	2	60.77 ± 0.63
	6	91	1:2	16	3	74.18 ± 0.13
	7	72	1:5	50	1	44.97 ± 1.22
	8	100	1:4	29	2	97.12 ± 0.72
	9	83	1:5	40	2	84.32 ± 0.37
Centroid search	10	91	1:4	31	2	84.98 ± 0.02
	11	89	1:4	34	2	82.20 ± 0.36
	12	91	1:3	28	2	76.57 ± 0.21
	13	90	1:3	29	2	76.85 ± 0.06

**Table 2:** Results of the second round of experiments.

Step	Experiment No.	Ethanol Concentration (%)	Solid–Liquid Ratio	Ultrasonic Time (min)	Number of Deacidification Cycles	Deacidification Rate (%)
Random search design	1	96	1:4	26	3	97.13 ± 0.04
	2	86	1:5	28	2	77.88 ± 0.46
	3	95	1:4	22	1	65.99 ± 0.50
	4	85	1:4	40	1	55.48 ± 0.41
	5	96	1:3	22	2	89.17 ± 0.01
	6	97	1:4	34	1	71.89 ± 0.30
	7	90	1:4	39	2	83.47 ± 0.82
Centroid search	8	95	1:4	29	2	89.96 ± 0.66
	9	96	1:4	27	2	93.51 ± 0.02
	10	94	1:4	31	2	88.78 ± 0.35
	11	93	1:4	30	2	83.88 ± 0.48

**Table 3:** Effect of deacidification method on physical and chemical quality of safflower seed oil.

Oil Sample Name	Acid Value (mg/g)	Deacidification Rate (%)	Peroxide Value (mmol/kg)	Anisidine Value	Total Oxidation Value
Crude oil	2.32 ± 0.02 ^a^	-	1.01 ± 0.04 ^b^	0.57 ± 0.01 ^b^	2.59 ± 0.09 ^b^
UAEE deacidification	0.10 ± 0.01 ^c^	97.13 ± 0.70 ^a^	0.96 ± 0.00 ^b^	0.63 ± 0.02 ^b^	2.56 ± 0.02 ^b^
Alkali refining deacidification	0.97 ± 0.05 ^b^	72.16 ± 0.13 ^b^	1.50 ± 0.09 ^a^	1.00 ± 0.04 ^a^	3.99 ± 0.15 ^a^

Note: Letters a–c indicate that different letters in a column differ significantly (*p* < 0.05). Each value is expressed as mean ± standard deviation (*n* = 3).

**Table 4:** Effect of deacidification methods on fatty acids of safflower seed oil.

Oil Sample Name	Linoleic Acid (%)	Oleic Acid (%)	Palmitic Acid (%)	Linolenic Acid (%)	Stearic Acid (%)
Crude oil	72.89 ± 0.28 ^b^	12.81 ± 0.15 ^b^	6.50 ± 0.04 ^a,b^	3.65 ± 0.04 ^b^	2.60 ± 0.08 ^b^
UAEE deacidification	73.78 ± 0.11 ^a^	13.08 ± 0.25 ^b^	6.27 ± 0.14 ^b^	4.11 ± 0.07 ^a^	2.76 ± 0.15 ^b^
Alkali refining deacidification	69.47 ± 0.22 ^c^	14.38 ± 0.03 ^a^	6.73 ± 0.03 ^a^	3.05 ± 0.00 ^c^	3.70 ± 0.05 ^a^

Note: Letters a–c indicate that different letters in a column differ significantly (*p* < 0.05). Each value is expressed as mean ± standard deviation (*n* = 3).

**Table 5:** Effects of different deacidification methods on lipid concomitant compounds of SSO.

Oil Sample Name	α-Tocopherol (mg/kg)	Total Phenols (mg/kg)	Sterols (mg/kg)
Crude oil	186.96 ± 0.88 ^a^	11.06 ± 0.16 ^a^	73.00 ± 2.65 ^a^
UAEE deacidification	177.30 ± 0.60 ^b^	9.12 ± 0.09 ^b^	66.00 ± 1.00 ^b^
Alkali refining deacidification	154.30 ± 0.36 ^c^	7.25 ± 0.05 ^c^	58.67 ± 1.53 ^c^

Note: Letters a–c indicate that different letters in a column differ significantly (*p* < 0.05). Each value is expressed as mean ± standard deviation (*n* = 3).

**Table 6:** Effect of deacidification methods on antioxidant activity of SSO in vitro.

Oil Sample Name	DPPH Radical Scavenging Capacity (μmol TE/100 g)	ABTS Radical Scavenging Capacity (μmol TE/100 g)
Crude oil	35.76 ± 0.09 ^a^	14.65 ± 0.03 ^a^
UAEE deacidification	35.30 ± 0.28 ^a^	14.19 ± 0.01 ^b^
Alkali refining deacidification	32.11 ± 0.28 ^b^	13.93 ± 0.12 ^b^

Note: Letters a,b indicate that different letters in a column differ significantly (*p* < 0.05). Each value is expressed as mean ± standard deviation (*n* = 3).

**Table 7:** Correlation analysis of physical and chemical quality of SSO samples.

	α-Tocopherol	Total Phenolics	Total Sterols	Acid Value	Peroxide Value	Anisidine Value	Total Oxidation Value
α-Tocopherol	1						
Total phenolics	0.972	1					
Total sterols	0.989	0.975 **	1				
Acid value	0.408	0.733	0.677	1			
Peroxide value	−0.948	−0.806	−0.819 *	−0.187	1		
Anisidine value	−0.982	−0.914 *	−0.939 **	−0.370	0.945 **	1	
Total oxidation value	−0.961	−0.574	−0.707	−0.250	0.996 **	0.971 **	1

Note: * indicates significant correlation (*p* < 0.05), ** indicates extremely significant correlation (*p* < 0.01).

**Table 8:** Correlation analysis between lipid concomitant compounds and antioxidant capacity in vitro.

	α-Tocopherol	Total Phenol	Total Sterols	ABTS Radical Scavenging Capacity	DPPH Radical Scavenging Capacity
α-Tocopherol	1				
Total phenol	0.972	1			
Total sterols	0.989	0.975 **	1		
ABTS radical scavenging capacity	0.950	0.932 **	0.867 *	1	
DPPH radical scavenging capacity	0.970	0.911 *	0.934 **	0.840 *	1

Note: * indicates significant correlation (*p* < 0.05), ** indicates extremely significant correlation (*p* < 0.01).

**Table 9:** Range of parameters to be optimized.

Number of Optimization Cycles	Factors to be Optimized	Ethanol Concentration (%)	Solid–Liquid Ratio	Ultrasonic Time (min)	Number of Deacidification Cycles
First round	Factor upper limit	100	1:1	50	3
	Lower limit of factor	70	1:5	10	1
Second round	Factor upper limit	100	1:3	40	3
	Lower limit of factor	85	1:5	20	1

## Sample Availability

Samples of the compounds are available from the authors.

## Abbreviations

UAEE	Ultrasonic-assisted ethanol extraction
RCO	Random centroid optimization
DPPH	2,2-Diphenyl-1-picrylhydrazyl
ABTS	2,2′-Azinobis (3-ethylbenzothiazoline-6-sulfonic acid)
SSO	Safflower seed oil
